# Effect of Hydroxyl Monomers on the Enzymatic Degradation of Poly(ethylene succinate), Poly(butylene succinate), and Poly(hexylene succinate)

**DOI:** 10.3390/polym10010090

**Published:** 2018-01-18

**Authors:** Zhenhui Bai, Yun Liu, Tingting Su, Zhanyong Wang

**Affiliations:** 1 College of Chemistry, Chemical Engineering and Environmental Engineering, Liaoning Shihua University, Fushun 113001, China; m182423647212@163.com; 2 College of Life S ciences and Oceanography, Shenzhen University, Shenzhen 518060, China; sunshine@szu.edu.cn

**Keywords:** poly(ethylene succinate), poly(butylene succinate), poly(hexylene succinate), cutinase, biodegradability

## Abstract

Poly(ethylene succinate) (PES), poly(butylene succinate) (PBS), and poly(hexylene succinate) (PHS), were synthesized using succinic acid and different dihydric alcohols as materials. Enzymatic degradability by cutinase of the three kinds of polyesters was studied, as well as their solid-state properties. The biodegradation behavior relied heavily on the distance between ester groups, crystallinity, and the hydrophilicity-hydrophobicity balance of polyester surfaces. The weight loss through degradation of the three kinds of polyesters with different hydroxyl monomers took place in the order PHS > PBS > PES. The degradation behavior of the polyesters before and after degradation was analyzed by scanning electron microscopy, differential scanning calorimetry, powder X-ray diffraction, Fourier transform infrared spectroscopy, gel permeation chromatography, and thermogravimetric analysis. The decrease in relative intensity at 1800–1650 estedpolyesters were degraded simultaneously. The frequencies of the crystalline and amorphous bands were almost identical before and after degradation. Thus, enzymatic degradation did not change the crystalline structure but destroyed it, and the degree of crystallinity markedly decreased. The molecular weight and polydispersity index only changed slightly. The thermal stability of the three kinds of polyesters decreased during enzymatic degradation.

## 1. Introduction

Given that energy and environmental problems are currently attracting considerable attention, the use of traditional and non-biodegradable plastics—Including polypropylene (PP), polyethylene (PE), and low-density PE—Is severely restricted. Thus, biodegradable polymers are increasingly receiving attention as potential candidates for green materials. Aliphatic polyesters exhibit remarkable physicochemical properties, mechanical properties, and processability, which are comparable with those of PP and PE. Polycaprolactone (PCL), poly(3-hydroxybutyrate) (PHB), poly(ethylene succinate) (PES), poly(butylene succinate) (PBS), poly(butylene adipate), poly(hexylene succinate) (PHS), and poly(hexylene adipate) are some of the most promising biodegradable polymer materials in the field [1,2,3,4,5]. Among them, PES, PBS, and PHS have favorable biodegradability and biocompatibility, and they are widely used in films; injection-molded products; and clothing, pharmaceutical, medical, and biomedical industries [6,7,8].

Biodegradability is an indispensable utility of aliphatic polyester materials. This feature has received extensive research attention, and is the basis of studying polymers of biological origin. A considerable number of factors have been found to affect the degradation rate of polyesters under enzymatic hydrolysis. For example, one critical factor in the chemical structure of the polymer is the distance between ester groups in polyesters [9,10,11,12,13,14]. Recently, it has been widely accepted that crystallinity has an effect on degradation rates [15,16]. Some hydrophilic end groups (carboxyl and hydroxyl) can also promote the degradation of polyesters. The surface hydrophilicity-hydrophobicity balance of polyesters is also an important factor in enzymatic degradation [12,17,18,19,20,21,22]. The spherulite size, lamellar structure, pH, temperature, and enzyme concentration may also play significant roles during enzymatic degradation [13,15].

The selection of depolymerase is a key factor in achieving a high degradation rate. Polyester films can be decomposed completely within a few hours with different sources of cutinase, whereas microorganisms and lipase require several days to degrade with relatively low degradation rates (approximately 50%) [23,24,25,26,27]. Maeda et al. observed that poly-butylene succinate-*co*-adipate films become obviously rough after 6 h of degradation using cutinase, which was purified from PBS-degrading enzymes [28]. Hu et al. degraded PBS using cutinase cloned from *Fusarium solani*; the polyester was degraded completely within 6 h [29]. Only a few researchers have studied the degradation of PES by enzymes [8,30]. In particular, data for PHS biodegradability has only been reported from degradation studies in river water [31].

Few systematic studies have compared the biodegradability of different polyesters. In the present study, we prepared three kinds of aliphatic polyesters and compared their degradation behavior. The effects of hydroxyl monomers on degradation were also discussed. Changes in film morphology, crystallinity, chemical groups, molecular weight, and thermal behavior of the three different kinds of polyesters after degradation were studied using scanning electron microscopy (SEM), differential scanning calorimetry (DSC), powder X-ray diffraction (XRD), Fourier transform infrared spectroscopy (FTIR), gel permeation chromatography (GPC), and thermogravimetric (TG) analysis.

## 2. Materials and Methods 

### 2.1. Materials

Three polyesters were synthesized from succinic acid and aliphatic diols with two, four, and six methylene groups. Succinic acid (purum 99.5%), eythylene glycol (purum, 99%,), 1,4-butanediol (purum, 98%), and 1,6-hexanediol (purum 98%) were purchased from the Aladdin Biochemical Company (Shanghai, China). Titanium isopropoxide (purum, 95%), used as a catalyst, and decahydronaphthalene (purum, 99%), used as a solvent, were also purchased from the Aladdin Biochemical Company (Shanghai, China). Cutinase was purified from zymotic fluid of recombinant *Pichia pastoris* containing a gene encoding cutinase from *F. solani* as described previously [23]. All reagents were of analytical grade.

### 2.2. Synthesis of Polyesters and Films

Synthesis of PES, PBS, and PHS followed the two stages of esterification and polycondensation [32]. In the first stage, a proper molar ratio of succinic acid and diol with different hydroxyl monomers was added to 60 mL of odecahydronaphthalene at 190 °C in a nitrogen atmosphere. Titanium isopropoxide (1/600 of the total molar mass of reactants) was used as a catalyst and added into a flask, undergoing stirring. In the second stage, polycondensation was completed at 230 °C for 12 h of reaction. The polyesters were then dissolved in chloroform and washed with methanol three times.

Polyester films (thickness of 0.1 mm) were prepared by hot pressing at *T* = *T*_m_ + 40 °C and then cold pressing at room temperature at 50 MPa for several minutes. To reach equilibrium crystallinity, the films were kept at room temperature for three weeks and then reserved in a dry desiccator [14].

### 2.3. Enzymatic Degradation

The polyester films with a size of 30 mm × 10 mm × 0.1 mm (length × width × thickness, about 130–150 mg) were incubated in a 20 mM Na_2_HPO_4_-NaH_2_PO_4_ buffer (pH 7.4) at 37 ± 0.1 °C in 10 mg·mL^−1^ cutinase. After degradation for different lengths of time, the films were carefully gathered and rigorously washed with distilled water. They were then dried under vacuum until their weight was at a constant. The weight loss ratio of the three kinds of polyesters was calculated as Equation (1):

(1) $$W_{loss}(\%)=\frac{W_{before}-W_{after}}{W_{before}}\times 100\%$$

where *W*_loss_ (%) is the weight loss ratio of the polyester films, *W*_before_ is the weight of the polyester films before degradation, and *W*_after_ is the weight of the polyester films after degradation. The degradation experiments were repeated six times for each specimen. The values of *W*_before_ and *W*_after_ were estimated six times.

### 2.4. SEM Observations

The morphology of pre- and post-degraded polyester films was observed using SEM (SU8010, Hitachi, Tokyo, Japan) at 20 kV acceleration. A thin gold layer was sprayed onto the surface of the films before testing.

### 2.5. DSC Analysis

DSC (TA Instruments, Q20, New Castle, DE, USA) was conducted for calorimetric and crystallization measurements. Approximately 7 mg of the sample was heated from 40 to 150 °C at 10 °C·min^–1^ and held for 5 min in a nitrogen atmosphere to erase its thermal history. The samples were then cooled to 40 °C at 10 °C·min^−1^. The second heating scans were performed under the same conditions to characterize the crystallinity and melting behavior.

### 2.6. XRD Analysis

XRD patterns were obtained with an S8 Tiger (Bruker, Karlsruhe, Germany) Advance diffractometer with Cu Kα (λ = 1.5418 Å, 40 kV, 40 mA) radiation in the scan range of 5°–60° with a step size of 0.02°.

### 2.7. FTIR Analysis

FTIR spectra were obtained using an FTIR spectrometer (Agilent Cary 660, Santa Clara, CA, USA) equipped with a slide-on ATR accessory (Agilent, Santa Clara, CA, USA). Sixteen scans were co-added from the range of 4000–400 cm^−1^ with a resolution of 2 cm^−1^.

### 2.8. Water Contact Angle (WCA)

The wettability of the three kinds of polyester films was determined by measuring the WCA at room temperature under static conditions using a contact angle meter (KRUSS, DSA100, Hamburg, Germany). The polymer films were washed in ethanol and deionized water several times. The contact angle was estimated five times.

### 2.9. GPC Analysis

GPC was used for the analysis of the average molecular weights and molecular weight distribution of the three kinds of polyesters before and after degradation. GPC was conducted with a Waters 1515 Isocratic HPLC Pump (Milford, MA, USA). A Waters 1515 refractive index detector was used with a temperature controller and tungsten lamp at 35 °C. Chloroform (1.5 mg·mL^−1^) was used as an eluent with 0.80 mL·min^−1^ flow at 35 bar. A Waters Styragel HT column was used, and different molecular masses of polystyrene were utilized as standard in the range of 2000–10,000 g·mol^−1^. 

### 2.10. TG Analysis

The thermal degradation behavior of the three kinds of polyesters was studied by TG analysis (TA Instruments, Q600, New Castle, DE, USA). About 10 mg of the sample was heated from room temperature to 500 °C at 10 °C·min^−1^ in a nitrogen atmosphere (30 mL·min^−1^).

## 3. Results and Discussion

### 3.1. Enzymatic Degradation

**Figure 1:**
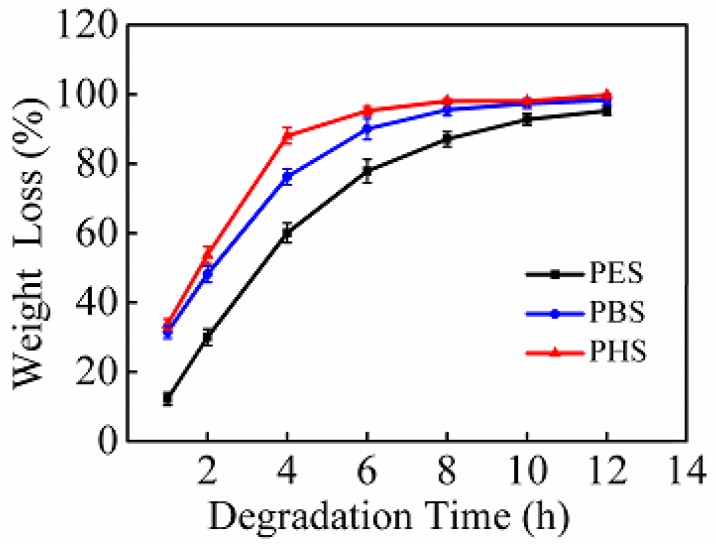
Weight loss of the three kinds of polyester films degraded for different lengths of time by cutinase.

Figure 1 illustrates the weight loss of the three kinds of succinate polyesters with prolonged degradation time. The weight loss was 60.1%, 76.3%, and 88.1% for PES, PBS, and PHS after degradation for 4 h, respectively. The weight loss of PES, PBS, and PHS reached 95.3%, 98.4%, and 99.8% after 12 h of enzymatic hydrolysis, respectively. The polyesters underwent two stages of weight loss: a fast stage (from 0 to 4 h) and a slow stage (from 4 to 12 h). In the fast stage, the weight loss of the polyester films increased rapidly. The rate of biodegradation is related to the slope of the degradation curve. The slope of the biodegradation curve (from 0 to 4 h) of PHS was 18.0, which was greater than that of PES and PBS, which were 15.8 and 14.8, respectively. Obviously, PHS exhibited the maximum rate of biodegradation in the fast stage. In the second stage, weight loss proceeded continuously but at a slow rate. With the attachment of enzymes to the polyester surfaces, the macromolecules changed into oligomers and segments of low molecular weight in the first degradation. The low rate in the second stage was due to the removal of oligomers and segments from the surface of polyesters [8].

As shown in Figure 1, the weight loss of the three kinds of polyesters was in the order PHS > PBS > PES. Considering that the degree of enzymatic degradation strongly depended on the distance between ester groups, hydrolysis occurred preferably at the ester groups with high methylene contents, instead of a random scission [9,10,11,12,13,14]. Similar conclusions were inferred by Rizzarelli et al. [14]; they degraded poly(butylene succinate-*co*-butylene sebacate) films and verified that enzymatic degradation has partial selectivity in the esterolysis reaction instead of a random breakdown. Fields and Bikiaris et al. both reported that aliphatic polyesters with six carbon atoms are most swiftly degraded, and biodegradability decreases with decreasing numbers of methylene groups between ester groups [8,12]. In our present work, PES, PBS, and PHS were derived from C_2_, C_4_, and C_6_ dihydric alcohols, respectively. Among them, PHS films carrying six carbon atoms exhibited the maximum weight loss. Compared with PHS, PBS containing four methylene groups exhibited the second greatest weight loss, whereas PES with two methylene groups showed the least loss. In addition to the distance between ester groups, the degree of enzymatic hydrolysis was also affected by other factors, such as crystallinity, hydrophilicity, and molecular weights, which will be discussed in the following section.

### 3.2. Morphological Observations

**Figure 2:**
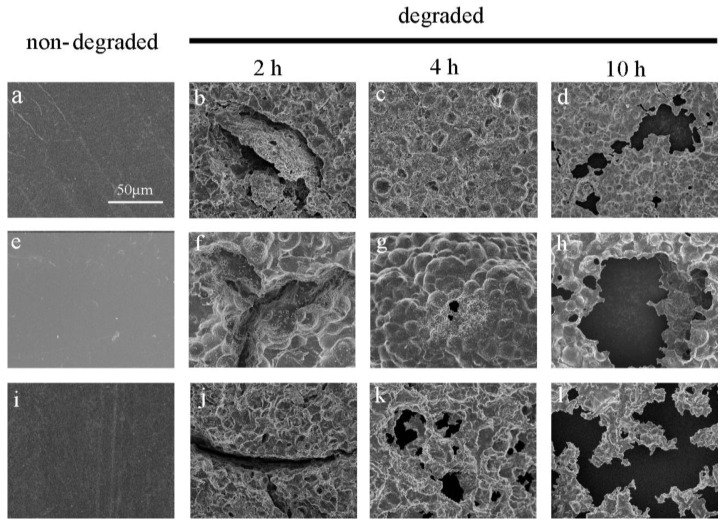
SEM images of the three kinds of polyester films degraded for different lengths of time. Poly(ethylene succinate) (PES): (**a**–**d**); poly(butylene succinate) (PBS): (**e**–**h**); and poly(hexylene succinate) (PHS): (**i**–**l**) degraded for 0, 2, 4 and 10 h.

**Figure 3:**
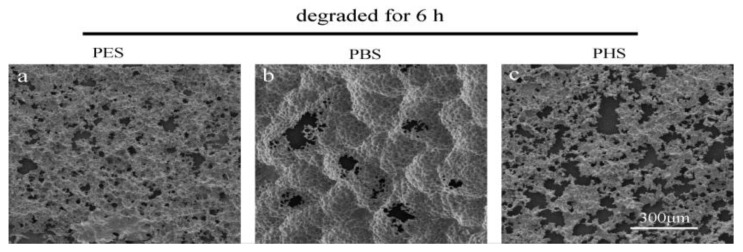
Panoramic SEM images of the three kinds of polyester films degraded for 6 h ((**a**)—PES; (**b**)—PBS; and (**c**)—PHS).

The surface morphology of the partially degraded films of the three kinds of polyesters was determined via SEM observations (see Figure 2 and Figure 3). Originally, the three kinds of polyester films demonstrated a homogeneous surface. The first signs of enzymatic hydrolysis were some cracks appearing on the surfaces of polyester films after 2 h of enzymatic erosion. Some holes arose and became increasingly deeper with increasing erosion time. The surface was irregular after degradation, lasting longer than 2 h. At the beginning of the PHS degradation process, some minor cavities and splits were observed, and they gradually increased with time until they were connected to one another. PES films showed similar behavior to PHS, but they degraded at a relatively low ratio. In contrast to PHS and PES, PBS films showed a spherulitic texture. They formed holes in several specific locations, which then became larger with increasing erosion time. This phenomenon was due to the enzymatic erosion at the center of the crystals and separation of cracks [8]. This difference in degradation behavior between PES and PBS could be due to several factors, including crystallinity, chemical composition, and physical characteristics [8,33]. Figure 3 also shows that PHS exhibited the highest degradability, and enzymatic hydrolysis occurred all over the surface of the samples simultaneously.

### 3.3. Crystalline Properties

**Figure 4:**
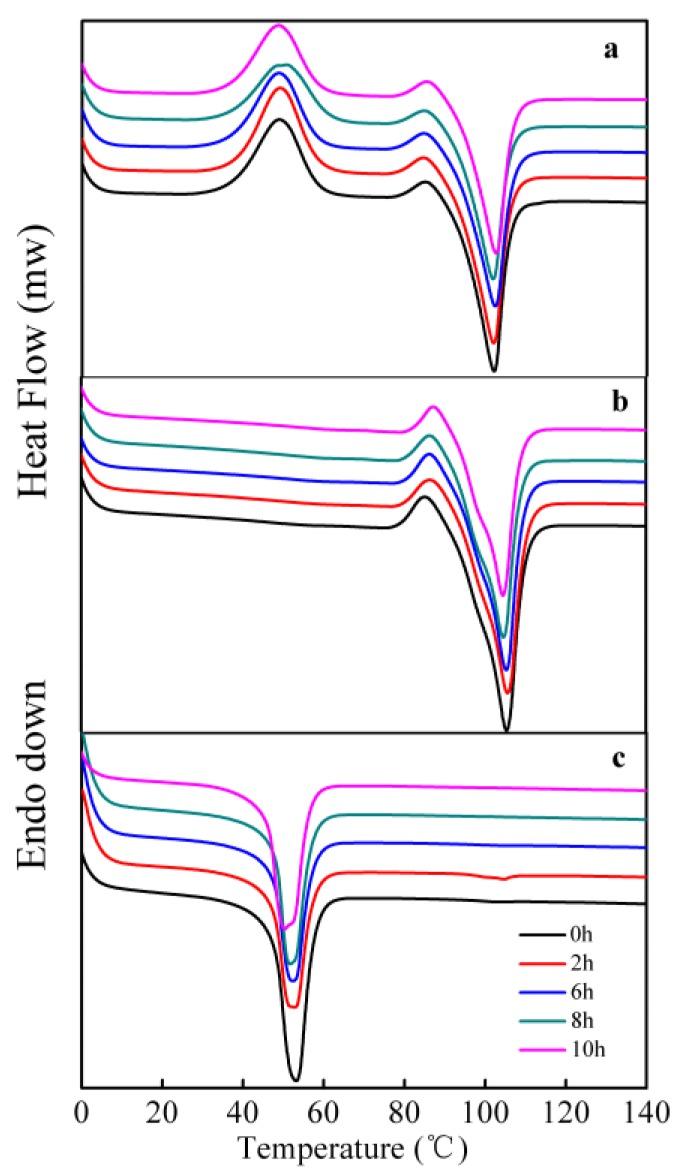
Differential scanning calorimetry (DSC) curves of the three kinds of polyester films degraded for different times ((**a**)—PES; (**b**)—PBS; and (**c**)—PHS).

Figure 4 and Table 1 show the changes in crystallinity of the three kinds of polyesters before and after degradation by cutinase. For all polyesters, the melting temperatures slightly decreased and the fusion and crystallinity heat markedly decreased with rising degradation time. The decrease in crystallinity was due to several factors, including a high initial crystallinity degree, the emergence of oligomers and molecular segments with low molecular weight, and water uptake [34]. The crystalline structure was damaged and the crystalline and non-crystalline regions of polyesters were degraded simultaneously by *Fusarium* sp. FS1301; these findings were consistent with results reported in the literature [4] and were certified by the following XRD and FTIR analysis. However, different findings were also reported by some researchers. They claimed that the crystallinity of polyesters increases with rising degradation time [8,35,36]. Abe et al. adopted depolymerase purified from *Pseudomonas pickettii* to degrade PHB, and they found that degradation occurs preferentially at the amorphous regions [15]. Furthermore, the specific degradability of cutinase cloned from *F. solani* might be responsible for the decrease in crystallinity during degradation.

**Figure 5:**
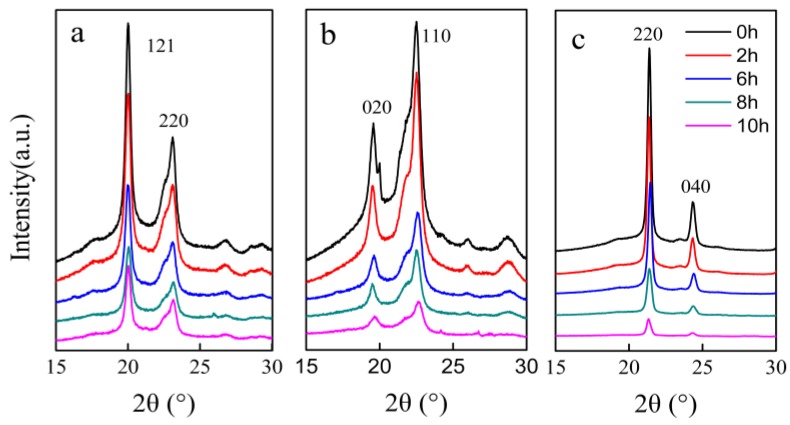
XRD patterns of the three kinds of polyester films degraded for different lengths of time ((**a**)—PES; (**b**)—PBS; and (**c**)—PHS).

The decrease in crystallinity of the three kinds of polyesters after degradation was clearly observed in XRD patterns (see Figure 5). The area of the diffraction peak gradually decreased with the extension of the enzymatic hydrolysis time in the three kinds of polyester specimens. However, the pattern shape and peak position were not altered after degradation. The same conclusion was found in other studies on PBS degradation [29].

The degree of crystallinity is closely related to degradability. The higher the crystallinity, the slower the degradation of polyesters [16]. The degrees of crystallinity of neat PES, PBS, and PHS were 64.4%, 48.8%, and 38.4%, respectively. The enzymatic degradability of the three kinds of polyesters took place in the order PHS > PBS > PES. In addition, the melting temperature and glass transition of polyester could also affect the rate of enzymatic degradation. It has been reported that the polyesters with lower melting temperatures and lower *T*_g_ degraded faster [38,39,40]. Among the three kinds of polyesters, PHS had the lowest melting point and lower *T*_g_ [6]. Thus, PHS (*X*_c_ = 42.1%, *T*_m_ = 52.2 °C, *T*_g_ = −40.0 °C) degraded faster than PES (*X*_c_ = 64.0%, *T*_m_ = 102.1 °C, *T*_g_ = −11.5 °C) and PBS (*X*_c_ = 48.2%, *T*_m_ = 105.1 °C, *T*_g_ = −44.0 °C) [8,38,41]. Furthermore, a broad cold crystallization peak was observed in PES specimens (Figure 4a); this peak was due to crystallization and recrystallization being remarkably slower for PES than for PBS and PHS [42].

**Figure 6:**
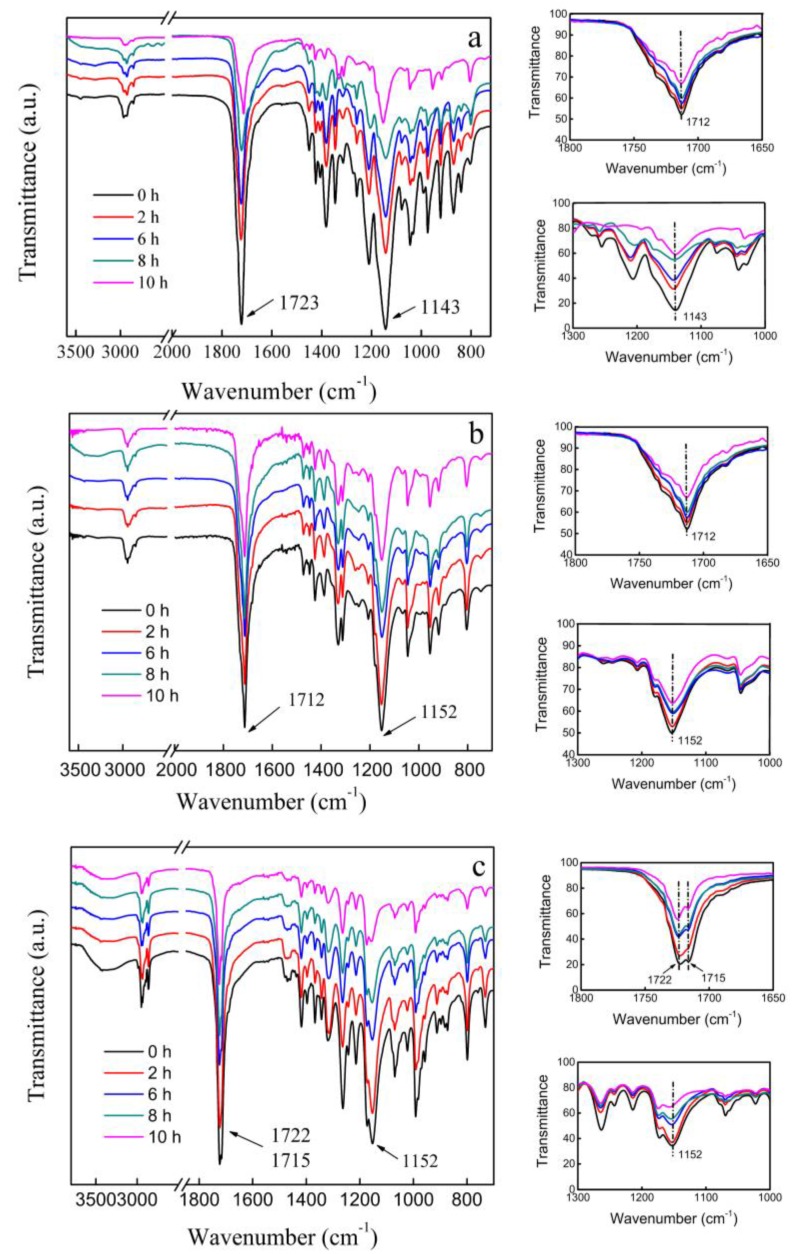
FTIR spectra of the three kinds of polyester films degraded for different lengths of time ((**a**)—PES; (**b**)—PBS; and (**c**)—PHS).

FTIR is highly sensitive to local structures and an effective instrument to distinguish between different polyester films. The FTIR spectra of all samples are shown in Figure 6 to analyze the changes after degradation. Compared with the initial spectra of the three polyesters, absorption peaks did not show apparent distinctions after erosion by cutinase. The primary peaks at around 1720 and 1150 cm^−1^ belonged to the stretching vibration of C=O and C–O–C, respectively. The FTIR spectra recorded in the 1800–1650 and 1300–1000 cm^−1^ regions are shown on the right of Figure 6. Peaks at 1723, 1712, and 1722 cm^−1^ were all assigned to the C=O stretching mode of the crystalline parts in the three kinds of polyesters [43,44,45,46]. These peaks were lower by about 25 cm^−1^ compared with the frequency of the stretching band of a free ester C=O group. These low frequency shifts were due to the crystalline packing [43]. The band at 1150 cm^−1^ probably consisted of two bands ascribed to the crystalline and amorphous states [43,44]. 

The bands in the 1800–1650 and 1300–1000 cm^−1^ regions revealed that the frequencies of the crystalline and amorphous bands were almost identical before and after degradation. These observations and the XRD results concluded that the structures of the crystalline states were highly similar before and after degradation. The C=O and C–O–C vibrations of the three kinds of polyester films before degradation were much more intense than those after degradation. The decrease in relative intensity of the two regions also demonstrated that enzymatic degradation simultaneously occurred at the crystalline and non-crystalline regions of polyester surfaces. Enzymatic degradation did not change the crystalline structure but destroyed it, thereby reducing crystallinity. These findings were consistent with the DSC and XRD measurements.

PES and PBS only had a single peak at 1720 cm^−1^, whereas PHS had double peaks at that wavenumber. The splitting of the C=O stretching vibration was due to the generation of the C–H–O hydrogen band between O atom of the C=O group and H atom of the –CH_2_– group [43,44]. Peaks at 1722 and 1715 cm^−1^ were due to the free carbonyl and hydrogen band, respectively. This result could be evidenced by the broad band above 3000 cm^−1^ (Figure 6c).

### 3.4. WCA Analysis

Besides the carbon chain length and degree of crystallinity, the hydrophilic-hydrophobic balance was also an important factor affecting the degradability of polyesters. The angles of PES, PBS, and PHS were 68° ± 0.2°, 74° ± 0.5°, and 78° ± 0.3°, respectively. Evidently, hydrophilicity progressively decreased as the carbon chain length increased. Several researchers illustrated that the hydrophilicity–hydrophobicity balance of polyesters exerts different effects when various enzymes are attached on the substrate surface [12,18,19,20,21,22]. Shirahama et al. studied the relationship between the hydrophilic properties of polyesters and degradability by different enzymes. Compared with hydrophilic polyesters, polyesters with more carbon atoms display a specific affinity for cholesterol esterase and lipase B and exhibit large weight loss and a fast degradation rate. However, opposing trends were observed when the substrate was degraded by lipase from *Rhizopus delemar* under the same conditions [47]. Unfortunately, the interaction between cutinase purified from *F. solani* and the hydrophilic polyester has not been investigated yet. In contrast to the degradation behavior of the three kinds of polyesters, cutinase exhibited high activity at the hydrophobic polyester surfaces. Therefore, degradability took place in the order PHS > PBS > PES depending on the different hydrophilic-hydrophobic properties. When the water-soluble enzymes came into contact with a hydrophobic surface, their active sites became completely approachable, and the enzyme assumed the active conformation [14].

### 3.5. Molecular Weight Analysis

Regarding the reliance of molecular weight on degradation rate, Li and Tokiwa et al. proved that the rate of degradation is independent from the molecular weight when the molecular weight exceeds 4000 g/mol [48,49]. The molecular weights of PES, PBS, and PHS were beyond 30,000 g/mol, so the effect of molecular weight on degradability was excluded in our present work. The molecular weights of our synthesized polyesters are listed in Table 2, which also shows the molecular weights and polydispersity indices of the three kinds of polyesters after degradation. Only minimal changes were found, which implied that degradation occurred homogeneously on the surface of the films. Moreover, the low-molecular-weight fragments of macromolecules did not accumulate, and their molecular weights remained unchanged. This result was in accordance with the enzymatic degradation of PCL and PHB [5,50].

### 3.6. TG Analysis

**Figure 7:**
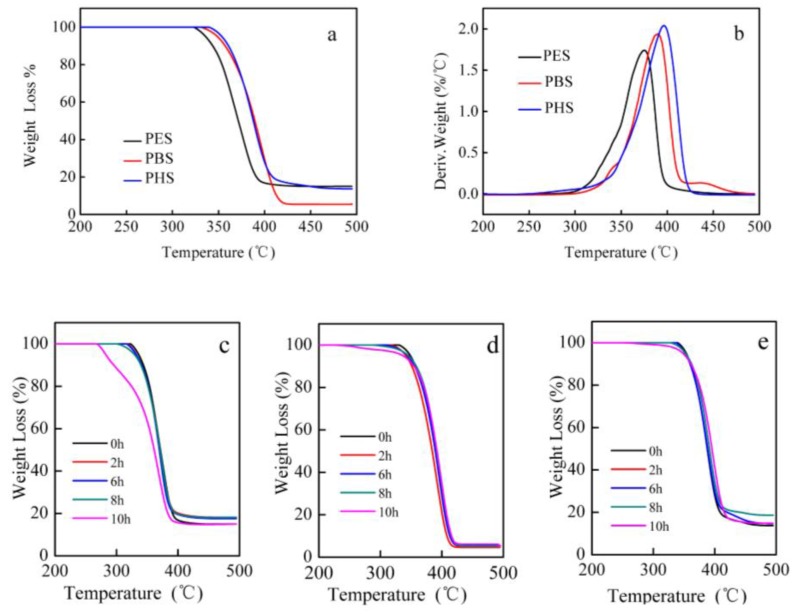
TG/DTG curves of the three kinds of polyester films ((**a**)—TG; (**b**)—DTG); TG curves of PES, PBS, and PHS degraded for different times ((**c**)—PES; (**d**)—PBS; and (**e**)—PHS).

Thermal degradation of PES, PBS, and PHS was studied and the TG and the derivative thermogravimetric (DTG) are shown in Figure 7. It can be seen from TG curves that all the polyesters decomposed in one stage. However, there were small divergences in the initial stage of decomposition in DTG curves. It showed that the polyesters decomposed followed two different mechanisms [51]. The initial decomposition (about 200–300 °C) was attributed to the volatilization of small molecules, including residual catalysts, succinic acid, and different dihydric alcohols. Only slight differences in the three kinds of polyesters could be seen in DTG curves. A random cleavage of the ester bond took place in the second stage (about 320–450 °C), which was due to the β-CH hydrogen transfer. Carboxylic end groups and vinyl groups were formed during this chain scission [52]. Bikiaris et al. prepared PES and PBS with the same molecular weight. They concluded that PBS had higher thermal stability due to having more methylene groups [53]. However, in our study, the temperature of the maximum rate was 374, 388, and 396 °C for PES, PBS, and PHS, respectively (Figure 7b). This was due to the fact that, along with the number of methylene groups, the molecular weight and the polydispersity of the polyesters also play a significant role in thermal stability [53]. PHS films with high molecular weight and narrow polydispersity had high thermal stability. In addition, when the polyesters were degraded for different lengths of time, they gradually disintegrated as degradation progressed (Figure 7c–e). These changes caused the thermal decomposition temperature to shift from a high point to a low one.

## 4. Conclusions

This work focused on the differences in enzymatic degradation among three kinds of aliphatic polyesters with various hydroxyl monomers. The biodegradability of the three kinds of polyesters took place in the order PHS > PBS > PES. The high weight loss was due to the high methylene contents between ester groups, low melting temperature, enhanced surface hydrophobicity, and low crystallinity. The characteristic crystalline and amorphous vibration modes were at 1720 and 1150 cm^−1^ for the three kinds of polyesters, respectively. The decrease in the FTIR band intensity demonstrated that degradation simultaneously occurred at crystalline and non-crystalline regions. DSC, XRD, and FTIR showed the decrease in the degree of crystallinity, which was due to the crystalline structure being destroyed after enzymatic degradation. GPC analysis showed slight changes in molecular weight and polydispersity of the three kinds of polyester. Low-molecular-weight fragments of macromolecules did not accumulate on polyester films, and they were completely removed in the second stage of enzymatic degradation. TG analysis clarified that the thermal decomposition temperature decreased for all polyesters with increasing degradation time.

## Figures and Tables

**Table 1:** Thermal properties of the three polyester films before and after degradation for different times.

Time (h)	PES			PBS			PHS		
	*T*_m_ (°C)	∆*H*_m_ (J/g)	*X*_c_ (%)	*T*_m_ (°C)	∆*H*_m_ (J/g)	*X*_c_ (%)	*T*_m_ (°C)	∆*H*_m_ (J/g)	*X*_c_ (%)
0	102.1 ± 0.8	49.2 ± 1.1	64.0 ± 1.4	105.1 ± 0.7	53.1 ± 1.5	48.2 ± 1.4	52.2 ± 0.8	57.0 ± 1.2	42.1 ± 0.9
2	101.9 ± 0.6	47.1 ± 1.2	62.1 ± 1.6	105.1 ± 0.8	51.9 ± 1.2	47.0 ± 1.1	52.0 ± 0.6	55.0 ± 1.6	40.8 ± 1.2
6	101.7 ± 0.6	45.8 ± 1.0	59.8 ± 1.3	105.0 ± 0.6	50.2 ± 1.7	45.9 ± 1.5	51.1 ± 0.6	54.2 ± 1.3	40.0 ± 1.0
8	101.0 ± 0.5	45.1 ± 1.2	59.2 ± 1.6	104.2 ± 0.4	46.8 ± 1.1	42.3 ± 1.0	51.1 ± 0.3	51.4 ± 2.2	38.1 ± 1.6
10	101.0 ± 0.5	42.8 ± 0.9	56.6 ± 1.2	104.1 ± 0.4	44.1 ± 1.5	39.8 ± 1.4	49.8 ± 0.3	50.0 ± 1.1	36.9 ± 0. 8

Time: referred to the degradation time of polyesters; *T*_m_: melting temperature; ∆*H*_m_: enthalpy of fusion. $X_{C}(\%)=\frac{\Delta H_{m}}{\Delta H_{m}^{0}}\times 100\%$,$\Delta H_{m}^{0}=76.4$, 110, and 135 J/g for PES, PBS, and PHS, respectively [37].

**Table 2:** Molecular weight and polydispersity index (${\bar{M}}_{w}$/${\bar{M}}_{n}$) of the three polyester films before and after degradation for different times.

Degradation Time (h)	PES		PBS		PHS	
	${\bar{M}}_{n}$ (g/mol)	${\bar{M}}_{w}$/${\bar{M}}_{n}$	${\bar{M}}_{n}$ (g/mol)	${\bar{M}}_{w}$/${\bar{M}}_{n}$	${\bar{M}}_{n}$ (g/mol)	${\bar{M}}_{w}$/${\bar{M}}_{n}$
0	53,086	8.15	36,657	2.75	86,043	1.81
2	52,765	8.13	36,147	2.71	85,817	1.79
6	54,972	8.10	36,792	2.67	88,245	1.75
8	58,494	8.06	38,154	2.59	92,046	1.72
10	62,517	8.03	41,528	2.57	95,314	1.70
